# Changes in the Endophytic Bacterial Community of *Brassica rapa* after Application of Systemic Insecticides

**DOI:** 10.3390/ijms242015306

**Published:** 2023-10-18

**Authors:** Md. Tareq Bin Salam, Ryota Kataoka

**Affiliations:** 1 Faculty of Life and Environmental Sciences, University of Yamanashi, Kofu 400-8510, Yamanashi, Japan; g21dtka3@yamanashi.ac.jp; 2 Soil, Water and Environment Discipline, Khulna University, Khulna 9208, Bangladesh

**Keywords:** endophytic bacteria, insecticides, polymerase chain reaction, restriction fragment length polymorphism, bacterial diversity, principal component analysis

## Abstract

Insecticides not only control target pests but also adversely affect non-target communities including humans, animals, and microbial communities in host plants and soils. The effect of insecticides on non-target communities, especially endophytic bacterial communities, remains poorly understood. Two phases of treatments were conducted to compare the trends in endophytic bacterial response after insecticide application. Endophytic bacteria were isolated at 2 and 4 weeks after germination. Most insecticide treatments showed a declining trend in bacterial diversity and abundance, whereas an increasing trend was observed in the control. Therefore, insecticide use negatively affected non-target endophytic bacterial communities. *Bacillus* spp. was mostly dominant in the early stage in both insecticide treatment and control groups. Nevertheless, in the matured stage, mostly bacteria including *Pseudomonas* spp., *Priestia* spp. were dominant in groups treated with high insecticide concentrations. Therefore, plants can regulate and moderate their microbiome during their lifecycle depending on surrounding environmental conditions.

## 1. Introduction

Endophytes are endosymbiotic microorganisms that colonize plants without causing any apparent effects in the host [1]. A wide variety of bacteria and fungi are considered as endophytes that play potential roles in the protection of their host from pest attacks by promoting the resistance capacity of the host against diseases [2,3]. Therefore, most endophytic bacteria are considered to benefit plants and can improve crop growth under normal or stress conditions [4]. Under stress conditions, they modulate phytohormone production, which enables their host to grow and adapt to adverse conditions [4]. Therefore, studies on the endophytic bacterial community are important to elucidate plant susceptibility in specific environments.

Several plants are subjected to pest infestations throughout their growth, which leads to poor yields or even death. According to The Food and Agriculture Organization (FAO), 20–40% of crops are damaged annually owing to different pest infestations, which accounts for a loss of USD 290 billion annually [5]. Pesticides are indiscriminately used in the agricultural field to protect crops and prevent losses. Several insecticides have already been registered on the United States Environmental Protection Agency list as neonicotinoid-based insecticides, including acetamiprid, imidacloprid, clothianidin, dinotefuran, and thiamethoxam [6]. Neonicotinoids strongly bind to nicotinic acetylcholine receptors in the central nervous system of insects, which excessively stimulates the nerve cells and causes death [7]. In particular, neonicotinoids are systemic in plants. Additionally, although acephate is an organophosphorus insecticide, it is highly systemic in plants [8]. Due to their systemic nature, these insecticides can be easily absorbed by plants through their root systems or leaves and translocated to all parts of the plants [9]. Therefore, plants can store these insecticides for a long time, which, in turn, causes continuous contamination of all plant tissues, from roots to leaves and flowers [10]. For instance, active residue of neonicotinoids can stay in plant tissues for up to 1 year in woody plants [11] and acephate can persist in plants with residual systemic activity of about 10–15 days when recommended dose was applied [12]. Hence, this long-term toxicity causes detrimental effects on endophytic communities of plants. The level of toxicity highly depends on the plant varieties, their growth stages, and the amount of insecticide application [9]. However, these insecticides not only kill target pests but also negatively affect non-target macro and microorganisms including honeybees, butterflies, and moths among others [13], owing to their distinctive traits. This raises a large concern for crop sustainability because pesticide use may lead to the development of a poor endophytic community in plants [14]. Since certain endophytic bacteria play a major role in nutrient cycling for supporting the growth of host plants, changes in the bacterial groups can considerably influence host plant growth and stress tolerance [15]. In this context, routine monitoring of endophytic diversity changes and endophytic community development over time is needed to ensure future crop sustainability. Although several studies have evaluated the impact of pesticides on soil biodiversity [16,17,18] and environmental risks [19,20], studies on changes in endophytic bacterial communities upon systemic insecticide application are limited. Therefore, the aims of this study are to evaluate the endophytic bacterial community response when different insecticides are applied to the plant. Moreover, we conducted dinotefuran inoculation experiments with different concentrations to determine the influence on endophytic bacteria.

## 2. Results

### 2.1. Changes in Bacterial Population and Diversity Caused by Insecticide Application

**Figure 1:**
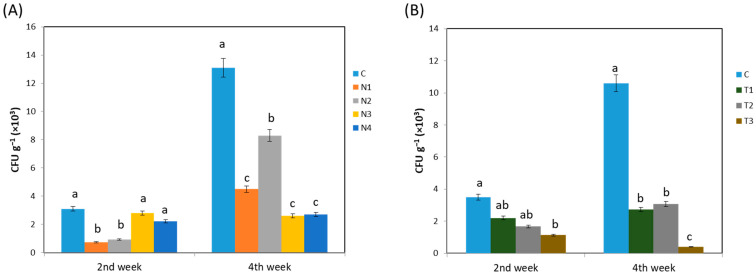
Effect of (**A**) different systemic insecticides; (**B**) different concentration of dinotefuran insecticide, on endophytic bacterial growth. Here, C, no insecticide condition; N1, imidacloprid treatment condition; N2, acetamiprid treatment condition; N3, acephate treatment condition; and N4, dinotefuran treatment condition as well as T1, single dose of dinotefuran; T2, double dose of dinotefuran; and T3, triple dose of dinotefuran. Values are mean ± standard deviation (n = 3). Different letter (a,b,c) above the bars indicates the level of significance among the treatments at *p* < 0.05 by Tukey–Kramer MRT test.

The effect of insecticide application on an endophytic bacterial population was investigated using the dilution procedure for each treatment at a definite time interval. The population enrichment of endophytic bacteria associated with insecticide application is presented in Figure 1. In experiment 1 (Figure 1A), the highest colony forming unit (CFU) was found for the control at both the 2nd and 4th weeks consecutively (*p* < 0.05). Among the treatment groups, N2 showed a higher CFU from the 2nd to 4th week when compared to that in the other treatments (*p* < 0.05). In contrast, N3 and N4 contained the lowest CFUs at the 4th week (*p* < 0.05), which were also lower than those at the 2nd week. In experiment 2 (Figure 1B), the findings were almost similar where the control showed the highest CFUs both at the 2nd and 4th weeks (*p* < 0.05). The CFUs reduced with an increasing dinotefuran concentration at both stages of *Brassica rapa*.

**Figure 2:**
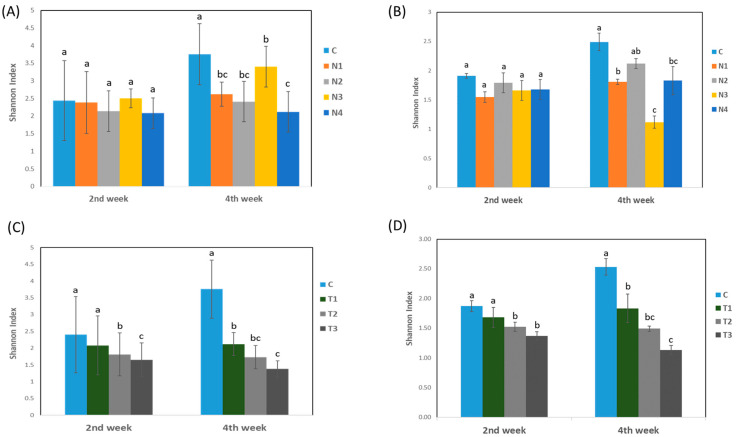
Endophytic bacterial diversity (Shannon–Weiner diversity index) (**A**) non-culturable index by using different systemic insecticides; (**B**) culturable index by using different systemic insecticides; (**C**) non-culturable index by using different concentration of dinotefuran insecticide; (**D**) culturable index by using different concentration of dinotefuran insecticide. Here, C, no insecticide condition; N1, imidacloprid treatment condition; N2, acetamiprid treatment condition; N3, acephate treatment condition; and N4, dinotefuran treatment condition as well as T1, single dose of dinotefuran; T2, double dose of dinotefuran; and T3, triple dose of dinotefuran. Values are mean ± standard deviation (n = 3). Different letter (a,b,c) above the bars indicates the level of significance among the treatments at *p* < 0.05 by Tukey–Kramer MRT, test.

The effect of insecticides on endophytic bacterial diversity was evaluated by analyzing the indices obtained from the Shannon–Weiner diversity index. A two-step experiment was performed to investigate the impact of insecticides on bacterial diversity. In experiment 1, changes in bacterial diversities were not significant among different treatments and the control at the 2nd week; however, the control showed significant improvement in diversity at the 4th week compared to that obtained after other treatments (Figure 2A,B). Almost similar findings were also observed in experiment 2, where increasing the doses of the insecticides reduced the diversity in the host plants over time (Figure 2C,D). Different findings were found during treatment at both stages of treatment. In experiment 1, the Shannon–Weiner diversity index for culturable microbes was higher at the 4th week in groups N2 and N1. Conversely, regarding the diversity index for non-culturable microbes, N3 at the 4th week showed higher diversity followed by N4 at the 4th week. Moreover, in experiment 2, the diversity reduced at the 4th week compared to that at the 2nd week when double and triple doses of dinotefuran (groups T2 and T3) were applied for both culturable and non-culturable microbes (Figure 2C,D). In contrast, the diversity increased sharply from week 2 to week 4 in group T1, although the increasing trend was lower than that of the control (Figure 2C,D).

### 2.2. Impacts of Insecticides on Endophytic Bacterial Abundance and Richness

To analyze the impact of insecticide treatment on bacterial abundance and richness, the taxonomic data of culturable and non-culturable microbes were analyzed. Based on restriction fragment length polymorphism (RFLP) analysis, the culturable bacteria were categorized into different groups. 

In experiment 1, nine different culturable endophytic bacterial species were found in the control of two-week-old *B. rapa* plants, which increased to 14 by the 4th week (Table 1A). Similarly, five and seven different endophytic bacterial species were found in N1 and N2 groups at the 2nd week, which increased to seven and 11 species at the 4th week, respectively. A contrasting trend was observed in groups N3 and N4. In groups N3 and N4, the number of endophytic bacterial species was lower at the 4th week than that at the 2nd week. For non-culturable bacterium isolation, almost 426 different species were identified at the 2nd and 4th weeks. Similar to the findings obtained for culturable bacteria, the control showed an increase in bacterial richness in non-culturable conditions (Table 1A). Notably, N3 showed an increase in abundance of endophytes to a greater extent from the 2nd to 4th week than that obtained in other groups (Table 1A). 

In experiment 2, the control showed the highest bacterial richness (both culturable and non-culturable bacteria) as the age of the host plant increased, similar to that in experiment 1 (Table 1B). It was also found that increasing the doses of dinotefuran reduced the bacterial richness (both culturable and non-culturable). Regarding bacterial richness of culturable microbes, five species were found in group T3 at the 2nd week, which decreased to four at the 4th week. However, in non-culturable conditions, both groups T2 and T3 did not show any increase in bacterial richness in host plants over time. Group T3 presented the lowest bacterial richness, which was 16 at the 2nd week and nine at the 4th week. Notably, group T1 showed an increase in bacterial richness with time (31 to 42). Several instances were found where several genera and species were shared between different treatments and different ages of plants. Figure S2 presents the bacterial richness, similarity, and differences among different treatments depending on the Amplicon Sequence Variants (ASVs) for non-culturable bacteria.

### 2.3. Taxonomic Changes after Insecticide Application

**Figure 3:**
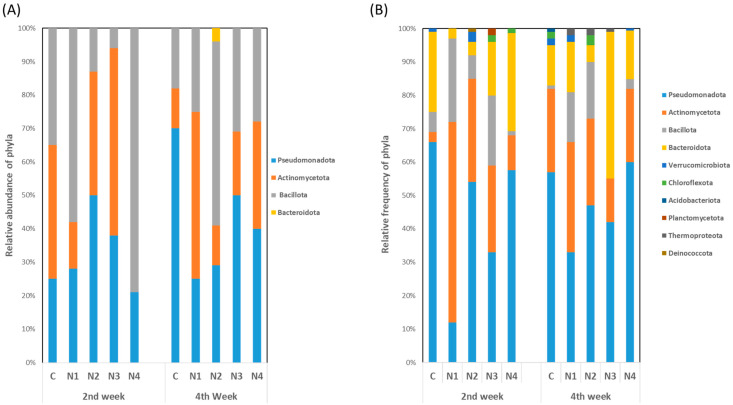
Relative frequency of phyla depending on (**A**) culturable isolates and (**B**) non-culturable ASVs. Here, C, no insecticide condition; N1, imidacloprid treatment condition; N2, acetamiprid treatment condition; N3, acephate treatment condition; and N4, dinotefuran treatment condition.

The identified culturable isolates and non-culturable Amplicon Sequence Variants (ASVs) revealed different taxonomic changes in different treatments over time. Figure 3 shows the relative abundance of phyla for each treatment in various treatment conditions. RFLP analysis of culturable bacteria revealed a total of three phyla at the 2nd week and four phyla at the 4th week (Figure 3A). Certain phyla, including Pseudomonadota, Actinomycetota, and Bacillota, were common both in control and insecticide treatment groups in *B. rapa* at both ages. Few differences were observed in the phylum abundance and distribution among treatments. In the 2nd week control group, the relative abundance of Actinomycetota (40%) was relatively higher, which was not prevalent at the 4th week. Subsequently, the abundance of Pseudomonadota (70%) was the highest in mature plants and dominated the overall diversity of the endophytic bacterial community. In contrast, Bacillota was dominant at the 2nd week in groups N1 and N4, and it was less dominant at plant maturity. Similar findings were observed in group N3. Here, Actinomycetota was dominant at the 2nd week whereas Pseudomonadota abundance increased at the 4th week. Notably, Bacteroidota was observed as a new phylum in group N4 at the 4th week, which was not recorded in any other treatments as well as the control.

Diverse findings were found for the phylum abundance of non-culturable microbes. Overall, 10 non-culturable phyla were identified during the growth of *B. rapa*. The number of phyla was higher in the control at the 4th week, where seven different phyla were recorded. At the early age of *B. rapa*, Pseudomonadota, Actinomycetota, Bacteroidota, and Bacillota were mostly found as common phyla whereas Pseudomonadota, Actinomycetota, and Bacteroidota were mostly dominant at the mature stage (Figure 3B). In addition, Thermoproteota appeared in most of the insecticide treatment conditions except dinotefuran. Notably, this phylum was absent in non-insecticide applied condition. 

**Figure 4:**
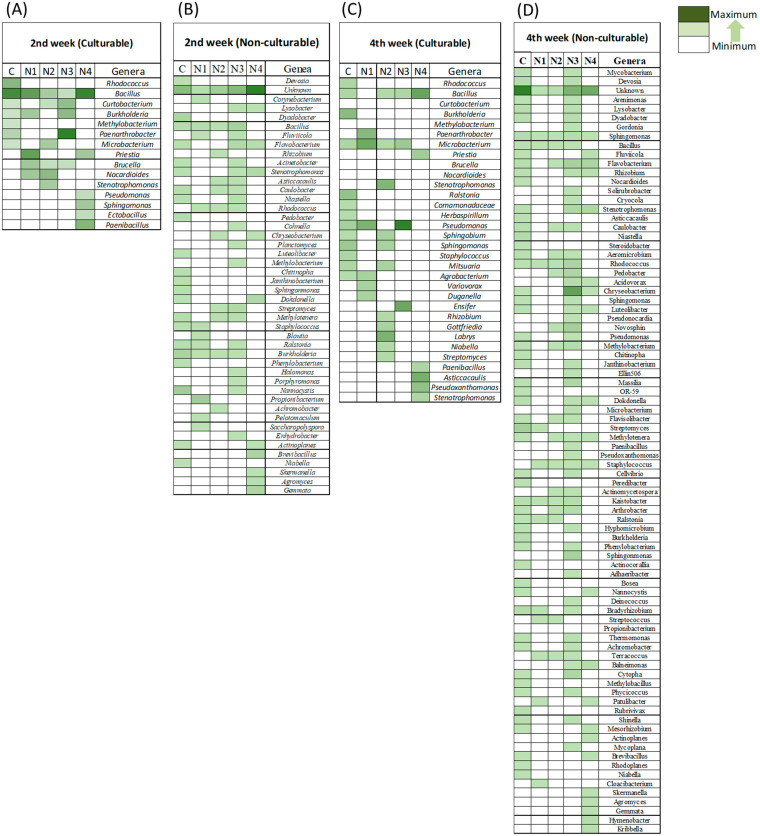
Heatmap illustrating the trend of (**A**) 2nd week culturable; (**B**) 2nd week non-culturable; (**C**) 4th week culturable; and (**D**) 4th week non-culturable bacteria from each genera. Here, C, no insecticide condition; N1, imidacloprid treatment condition; N2, acetamiprid treatment condition; N3, acephate treatment condition; and N4, dinotefuran treatment condition.

The heatmap (Figure 4) indicates the major changes in bacterial genera in different treatments over time. In culturable endophytic bacteria, *Bacillus* was the dominant genus in the control of groups N1 and N4 at the 2nd week (Figure 4A). In contrast, *Nocardioides* was dominant in group N2 and *Paenarthrobacter* was prevalent in group N3 at the 2nd week (Figure 4A). At the 4th week, *Burkholderia* was abundant in the control, whereas the abundance of the genus *Bacillus* was reduced (Figure 4C). In different insecticide treatment conditions, *Pseudomonas* was found to be dominant in both groups N1 and N3 (Figure 4C). Notably, *Labrys* was dominant at the 4th week in group N2 followed by *Stenotrophomonas*. Similar to that observed for week 2, *Bacillus* was also a major genus present at the 4th week in group N4 (Figure 4C).

The identification of non-culturable bacteria revealed that unknown genera were frequently abundant, in both insecticide treatment and control groups (Figure 4B,D). Furthermore, the genera *Nannocystis*, *Burkholderia*, and *Dyadobacter* were dominant in the control group at the 2nd week (Figure 4B). In the 2nd week, unlike in the control group, *Propionibacterium* and *Bacillus* were abundant in the group N1 instead of unknown genera. However, *Methylotenera* was also abundant in group N2, whereas *Bacillus* and *Brevibacillus* were abundant in groups N3 and N4, respectively. In the 4th week, *Streptomyces* was dominant in the control, whereas *Rhodococcus* and *Cloacibacterium* were also abundant in groups N1 and N2, respectively (Figure 4D). In contrast, *Flavobacterium* was identified as a common genus both in groups N3 and N4. The heatmap (Figure 4) revealed that the control group contained a larger number of endophytic genera and abundance than those in the insecticide-treated groups at both ages of *B. rapa*. Accordingly, the heatmap showed that the number of genera was higher in the control at both ages of the plants than that in the insecticide-treated groups. In total, 23 different genera were recognized in the control at the 2nd week, which increased to 55 at the 4th week. Although the insecticide treatment groups also showed an enrichment in endophytic bacterial genera from the 2nd to 4th week, these numbers were lower than that of the control at each week. 

Table S2 presents the changes in the frequency of endophytic bacteria among culturable microbes in groups T1, T2, and T3 over time. At the 2nd week, mostly *Bacillus* and *Paenibacillus* species were dominant in dinotefuran treatment and control conditions. An exception was observed in group T3, where *Margalitia* along with *Nocardioides* were mostly abundant instead of *Bacillus*. Additionally, at the 4th week, *Bacillus* species were dominant in the control group and group T1. In contrast, when relatively higher doses were applied, *Priestia* was dominant in group T2 and *Pseudomonas* represented a major member in group T3. The Venn diagram (Figure S2) presents the bacterial richness of non-culturable microbes after treatment with different doses of dinotefuran and illustrates that the control always contained a larger number of endophytic bacteria than that in the dinotefuran treatment groups, although they may share few common species.

### 2.4. Multivariate Analysis to Assess the Impact of Insecticides on Endophytic Bacterial Community

**Figure 5:**
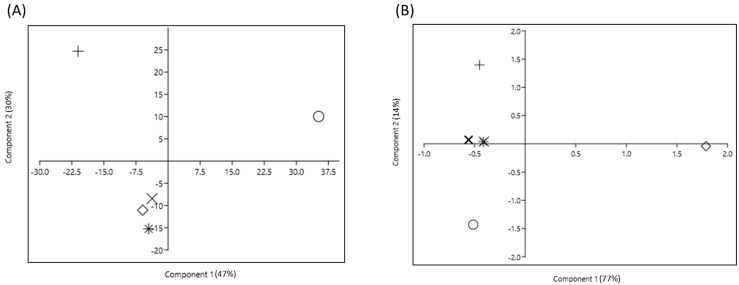
Principal component analysis (PCA), showing the microbial diversity due to different systemic insecticides’ application at (**A**) 2nd week; (**B**) 4th week based on ASVs. Here, +, no insecticide condition, *, imidocloprid treatment condition; ×, acetamiprid treatment condition; ◊, acephate treatment condition; and O, dinotefuran treatment condition.

**Figure 6:**
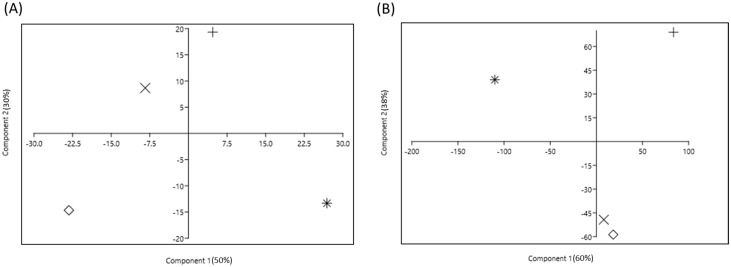
Principal component analysis (PCA), showing the microbial diversity due to different doses of dinotefuran application at (**A**) 2nd week; (**B**) 4th week based on ASVs. Here, +, no dinotefuran condition, *, single dose of dinotefuran; ×, double dose of dinotefuran; and ◊, triple dose of dinotefuran.

The principal component analysis (PCA) of the ASVs segregated the treatments into different coordinates. To assess the impact of insecticides on the endophytic bacterial community, four plot analyses were performed (Figure 5 and Figure 6). Figure 5 describes the impact of different insecticides on the endophytic bacterial community and Figure 6 elaborates the effect of different doses of dinotefuran on the endophytic bacterial community at the 2nd and 4th weeks. The control was positioned at considerable distances from all other insecticide treatment groups both at the 2nd and 4th weeks, which signified that insecticide use altered the endophytic bacterial community (Figure 5). Moreover, the groups N1, N2, and N3 were placed close to each other, which indicated a similar endophytic bacterial community (Figure 5A). A similar trend was observed in Figure 5B, where groups N1 and N2 showed a similar endophytic community, group N3 was separated broadly and developed a different endophytic bacterial community unlike that in the 2nd week. The heatmap (Figure 4) also presented the same findings as seen in the PCA plot separation. However, the principal components on scale 1 accounted for 47% variability in the 2nd week and 77% variability in the 4th week between the control and insecticide treatments combined with 30% variability in the 2nd week and 14% variability in the 4th week of principal components on scale 2. In experiment 2, the control also maintained a significant distance from all treatments at both the 2nd and 4th weeks (Figure 6). The findings suggest that treatment with different doses of dinotefuran had a significant effect on the endophytic bacterial community. In the 2nd week (Figure 6A), the principal components on scale 1 were responsible for 50% variability which separated the T2 and T3 groups broadly. In contrast, the 30% variability of principal components on scale 2 separated the T3 group largely. In contrast, in the 4th week, clustering was observed between groups T2 and T3 (Figure 6B), which indicated a similar endophytic community between these two treatments. However, the 77% variability in the principal component of scale 1 signified a higher separation between the control and different doses of dinotefuran treatment in plot segmentation, especially the groups T2 and T3. This result reflected that *B. rapa* plants treated with increasing doses of insecticides possess similar types of endophytes at maturity.

## 3. Discussion

In this study, the effects of four different insecticides and the impact of different concentrations of dinotefuran on the diversity and abundance of endophytic bacteria in host plants over a period of 4 weeks were investigated. The impacts of using four insecticides on endophytic bacterial diversity were not as evident at the 2nd week as they were at the 4th week. These findings indicated that synthetic insecticides negatively affected the enrichment of bacterial communities at a mature stage of plants. This is because we found a reduced bacterial richness in plants at maturity after insecticide treatment compared to that in the control of both the culturable and non-culturable groups. Additionally, increasing the doses of the insecticides resulted in significantly lower enrichment of bacterial diversity and richness in both stages of host plants. Therefore, the overuse of pesticides negatively affected the endophytic bacterial community development at the beginning of host plant life. This inhibitory effect associated with systemic insecticide treatment has been similarly reported in previous studies [21,22]. The continuous changes in communities of culturable and non-cultivable endophytic bacteria indicate an interaction between the host and the endophytic bacteria. Campisano et al. [23] stated that the development of bacterial endophytic communities in grapevines largely depends on pest management. In their experiment, a larger number of plant growth-promoting bacteria was observed after use of an integrated pest management system. Stuart A.K. et al. [14] conducted research on the impacts of pesticide use on endophytic fungal communities and revealed that agrochemicals significantly decrease the endophytic fungal diversity, richness, and evenness in conventional farming plots. Similarly, Win et al. [24] reported that in tea plant xylem, bark, and leaves, the colonization frequency of endophytic fungi is lower in a pesticide-treated plot than that in a plot without treatment. These studies support our findings, although they did not find any data on changes in microbial diversity related to host plant growth. We address this major knowledge gap in our study. In our study, we evaluated the endophytic bacterial diversity, richness, abundance, and community development at different growth stages of the host plants, which provides new insights in the field of endophytic research.

Different factors may be responsible for the reduced endophytic bacterial community richness observed in our experiment. Moulas et al. [25] claimed that the application strategy might affect the phyllosphere community. In their experiment, they used two systemic insecticides (imidacloprid and metalaxyl) and analyzed their effects on the phyllosphere community of pepper plants using different application methods (foliage and soil drenching). In their study, both application methods have been shown to negatively affect the phyllosphere community development, especially soil application, which causes more adverse impacts on bacterial community enrichment [25]. In our study, we performed soil drenching similar to that in the study of Moulas et al. [25]. Therefore, the application strategy may represent a potential factor affecting the reduced endophytic bacterial community development observed in our study. In nature, systemic insecticides can be easily taken up by plants and stored in their vessels [6]. Usually, the xylem and phloem vessels are responsible for translocating systemic insecticides inside plants after absorbing them from soil; however, the mechanism of exchange between xylem and phloem is poorly understood at present [26]. Generally, plants show changes in the endophytic community according to the surrounding conditions [27] and growth stages [28]. Plants provide shelter to certain groups of microorganisms that not only support their vegetative growth, but also provide support under stress conditions [29]. When plants absorb systemic pesticides, their internal microbiome may be adversely affected [30]. Once a plant is affected, the effects persist throughout its lifecycle. This may represent another reason for poor endophytic bacterial development in the insecticide treatment groups of the present study, which was more prominent at maturity. Unfortunately, the relationship among systemic insecticide uptake, translocation, and endophytic community enrichment is poorly understood. Further research is necessary to address this knowledge gap for elucidating the impact of pesticides on endophytic community development. 

In our second experiment, we identified the endophytic bacterial changes due to dinotefuran application at different doses. Dinotefuran, a systemic insecticide, can be transported to different plant parts [31,32]. Several studies have reported the environmental risk and eco-toxicity of dinotefuran in plants [33]. Yu et al. [34] recently reported that dinotefuran negatively affects the soil microbial community. In contrast, the impact of dinotefuran use on the endophytic microbial community remains unknown, which was explored in the present study. The increasing doses of dinotefuran reduced the endophytic bacterial diversity at the early stage of plant life, which was more prominent at maturity. The overuse of systemic insecticides can negatively affect the endophytic bacterial community, which supports the findings of Zaller and Brühl [35], who reported the negative impact of pesticide overuse on agroecosystems. In addition, Muturi et al. [22] reported that overuse of systemic pesticides can affect the non-target microbial communities in aquatic ecosystems by altering the physical and chemical conditions of the habitats. 

According to multivariate analysis, systemic insecticide application affected the overall endophytic bacterial community development in both experiments. PCA plot separation of different insecticides indicated that the control always contains an indigenous bacterial community at both stages (2nd and 4th weeks) of plant life. Through NGS data, we observed a higher number of phyla dominance in non-insecticide applied conditions at the matured stage of the plants. Moreover, distinct differences were perceived in genus level between insecticide applied and non-applied conditions at both culturable and non-culturable conditions. Bacterial genera such as *Sphingomonas*, *Streptomyces*, and *Lysobacter* were prominent in the control group and were rarely found in the insecticide treatment groups at plant maturity. These bacteria play major roles in the survival of plants in stress conditions by providing essential phytohormones and enzymes [36,37]. Conversely, insecticide treatment supported the growth of insecticide-degrading bacteria such as *Rhodococcus, Brucella*, and *Pseudomonas* [38,39]. The increase in dose of dinotefuran also promoted the growth of insecticide-degrading bacteria such as *Pseudomonas* and *Priestia* among others. Only *Pseudomonas* sp. can degrade a series of plant systemic pesticides such as imidacloprid [40], acetamiprid [41], thiamethoxam [42], clothianidin [43], etc. We also found *Rhodococcus* and *Brucella* to be the major genera in the early stages of plant life in most of the insecticide treatment conditions. These bacteria have the capability to degrade systemic pesticides such as acetamiprid [44] and dimethoate [39]. Changes in microbial diversity and abundance due to pesticide exposure can exert toxic effects in certain groups of non-target microorganisms, resulting in lower microbial diversity, whereas certain pesticide-tolerant microbes show increased growth owing to a decrease in competition for space and nutrients [16]. Moreover, some bacteria can utilize pesticides as a carbon and nitrogen source and consequently benefit from pesticide exposure [45]. Therefore, this may explain the reason for the presence of pesticide-degrading bacteria as dominant genera in different treatment groups, even though the overall diversity was reduced.

## 4. Materials and Methods

### 4.1. Experimental Design

Pot experiments were conducted to analyze the effects of insecticides on the endophytic bacterial community. A Gray Lowland soil was used as the growth substrate for the target plants, and it was collected from the University of Yamanashi, Japan research farm area (N35.604073, E138.578506). Gray lowland soils are formed on Holocence alluvial plains and consist of very deep well drained soils [46]. These soils are characterized by a gray or gray–brown color with poor nitrogen concentration. Here, NH_4_-N concentration is <1 mg kg^−1^ and NO_3_-N concentration is 25.6 mg kg^−1^ (personal investigation). The pH of the soil is 6.6 (H_2_O) along with 15.2 meq 100 g^−1^ cation exchange capacity (personal investigation). Additionally, the phosphorous concentration in the soil is 190 mg kg^−1^ whereas the water holding capacity (WHC) of the soil is 42% (personal investigation). All soil samples were air dried and filtered through a 2.0 mm mesh sieve for homogenization before the experiment. In each case, a 300 gm soil sample was taken from each pot. *Brassica rapa* var. *perviridis* (Atariya Holdings Co. Ltd., Chiba, Japan), local name Komatsuna or Japanese mustard spinach, was selected as the target plant for isolating endophytic bacteria, and they were grown in Neubauer pots (size, 500 mL). Komatsuna originates from Japan and is commercially cultivated in Japan as a popular leafy vegetables that can be cooked and eaten in various ways [47]. For growing *B. rapa*, the seeds were surface sterilized by washing with 70% ethanol for 1 min and 1% sodium hypochlorite (NaOCl) for 2 min. Subsequently, the seeds were washed and rinsed with sterilized distilled water (SDW) five times. The sterilized seeds were placed in wet Petri dishes for germination at room temperature (25 ± 2 °C). After germination, the seeds were transferred to experimental pots. Three seeds were sown in each pot. Since the experiment was conducted in a climate room set at 27 ± 1 °C, the water content of the soil was maintained at 50% of their water holding capacity, except on sowing days. In addition, the highest nutritional contents of Komatsuna were reported as 150 mg kg^−1^ nitrogen (N) concentration in the soil [48]. By considering the N status in Gray Lowland soils, 100 mg kg^−1^ N application is enough for the best growth of Komatsuna (personal investigation). This is why each pot was fertilized with a nitrogen (N) concentration of 100 mg kg^−1^ using Hyponex solution (N:P:K ratio of 6:10:5; HYPONeX JAPAN, Osaka, Japan) before transplanting the seedlings. The plant samples were collected consecutively at the second (early stage) and fourth weeks (matured stage) for isolating endophytic bacteria. The whole experiment was performed in two phases. A complete randomized design was followed for both phase experiments.

Experiment 1: We applied four different insecticides including imidacloprid, acetamiprid, acephate, and dinotefuran separately to the individual experimental pots. The application rates were 0.4 mg kg^−1^ for imidacloprid, 0.6 mg kg^−1^ for acetamiprid, 2.5 mg kg^−1^ for acephate, and 0.6 mg kg^−1^ for dinotefuran. The concentrations were calculated as per the recommended doses for application of the selected insecticides in Japan. The different insecticide treatments are presented as follows: N1, imidacloprid treatment condition; N2, acetamiprid treatment condition; N3, acephate treatment condition; and N4, dinotefuran treatment condition.

Experiment 2: We also evaluated the changes in the endophytic bacterial community based on application of different doses of insecticide. The treatments are presented as T1, single dose (0.6 mg kg^−1^) of dinotefuran; T2, double dose of dinotefuran; and T3, triple dose of dinotefuran. The application technique and frequency were the same as those in experiment 1. In both cases, no insecticide application was undertaken for the control (C) and all treatments along with control were performed in triplicate. Figure S1 presents the final layout of the experiment.

### 4.2. Plant Sample Preparation for Endophytic Bacteria

Plant samples were uprooted and washed in a running flow of water to remove the soil and other debris from the plant parts. Whole plant samples were kept at room temperature and the leaves were removed from the plant samples. The roots, shoots, and stems were further processed for isolating endophytic bacteria. The samples were then cut into 2–3 mm discs using aseptic scissors. All disc samples were surface sterilized using 70% ethanol for 1 min and 1% NaOCl for 2 min. Subsequently, the samples were washed with SDW five times. For confirmation of surface sterilization, the discs were embedded into the agar plate (2% agar) and monitored for microbial growth. No microbial growth was observed. After verifying the success of surface sterilization, half of the samples were taken for isolating culturable endophytic bacteria and the remaining half were stored at −80 °C for detecting non-culturable endophytic bacteria via next-generation sequencing.

### 4.3. Isolation of Culturable Endophytic Bacteria

To isolate culturable endophytic bacteria, 0.5 gm surface-disinfected plant samples were mashed in 4.5 mL of SDW using a sterilized mortar and pestle. In total, a 50 µL aliquot was poured over potato dextrose agar (Oxoid Ltd., Basingstoke, UK) plates (pH = 7) and spread equally. All plates were incubated for 3–4 d at 25 °C for colony observation. After distinct colonies appeared, the colony-forming units (CFUs) were calculated. Regarding pure culture isolation, colonies were selected based on the morphology of each colony evaluated by the naked eye. Morphologically similar colonies were counted as a single colony. The colonies were stored in a stock at −80 °C in 20% glycerol (in 0.8% NaCl *w*/*v*) solution.

### 4.4. DNA Extraction and PCR Amplification

Sterilized toothpicks were used to collect small amounts of bacterial colonies from the growing edge of colonies of each strain, which were added to the polymerase chain reaction (PCR) mixture. The PCR mixture was prepared by adding 1 µL of 341F (5′-CCTACGGGAGGCAGCAG-3′) and 1541R (5′-AAGGAGGTGATCCAGCC-3′), 9.5 µL of nuclease-free water, and 12.5 µL of GoTaq Green Master Mix. The 16S rRNA was amplified by following the PCR program: 94 °C for 5 min; 35 cycles at 94 °C; 58 °C for 30 s; 72 °C for 30 s; and extension of 72 °C for 7 min. Primer selection was dependent on the most suitable sequence length of culturable and non-culturable bacterium analysis. Further, gel electrophoresis was performed to verify the success of DNA extraction. 

### 4.5. PCR-Restriction Fragment Length Polymorphism (RFLP) Determination

To determine the richness of culturable bacteria, selected strains were further tested for their genotype using two restriction enzymes, Hae-III and Hha-I (Takara Biotechnology Co. Ltd., Shiga, Japan). In total, 2.5 µL of amplified nucleotide sample of each strain was mixed with each enzyme (0.5 µL), buffer (1 µL), and SDW (8.5 µL) in PCR tubes. All examined tubes were incubated at 37 °C for 60 min. After incubation, 1.25 µL of 10× loading buffer (Takara Biotechnology, Shiga, Japan) was added in the mixture. Subsequently, all mixtures were poured into the well of 2% agarose gel in TAE buffer and electrophoresed for 25 min. Finally, the electrophoresis bands were compared with a 100 bp DNA ladder after staining.

### 4.6. The 16S rRNA Gene Sequencing of Culturable Bacteria

The bacterial types were enumerated based on the similarities among strains determined via polymerase chain reaction–restriction fragment length polymorphism (PCR-RFLP). The direct sequencing method was followed to sequence the amplified nucleotides. The basic local alignment search tool (BLAST) searches in the database of GenBank (NCBI) were performed to identify the isolates. The identities of the isolates were confirmed by calculating the proportional similarities (>97%) using the reference list of NCBI BLAST search results (https://blast.ncbi.nlm, accessed on 25 September 2023). All sequence data were submitted in the DNA Data Bank of Japan (http://getentry.ddbj.nig.ac.jp/, accessed on 25 September 2023) under the accession numbers LC773881–LC773465 for experiment-1 and (Table S1) and LC773429–LC773486 for experiment-2 (Table S2).

### 4.7. Identification of Non-Culturable Endophytic Bacteria

The surface-sterilized plant samples were further used to isolate DNA using the FastDNA^TM^ Spin Kit for Environmental Sample (MP Biomedicals, Irvine, CA, USA) by following the manufacturer’s instructions. DNA extraction was verified via 2% gel electrophoresis and the DNA concentration was measured using a nano-spectrophotometer (ng µL^−1^). The DNA samples were extracted and normalized using 16S rRNA gene sequencing. Specific primers 515F (5′-GTGCCAGCMGCCGCGGTAA-3′) and 806R (5′-GGACTACHVGGGTWTCTAAT-3′) were used for the PCR-amplification of the V4 region of the bacterial 16S rRNA gene. Phusion^®^ High-Fidelity PCR Master Mix (New England Biolabs, Ipswich, MA, USA) was used to perform the PCR reactions. A 1× loading buffer (containing SYB green) was mixed with PCR products in the same amount for PCR quantification and qualification; additionally, electrophoresis was performed in 2% agarose gel for detection. Sequencing was performed by Bioengineering Lab (Sagamihara, Kanagawa, Japan) on the Illumina MiSeq platform by using the MiSeq system and the MiSeq Reagent Kit v3, Illumina 2 × 300 bp paired-end protocol. For ensuring reliability of the data, quality control was performed at each step of the procedure. DNA extraction without bacterial pellets under similar conditions was counted as the control. Based on the unique barcode, the pair-end reads were assigned and merged using FLASH (V1.2.7, https://ccb.jhu.edu/software/FLASH/, accessed on 25 March 2023) [49]. Data analysis was performed using the bacterial flora analysis pipeline QIIME software package (V1.7.0, https://qiime.org/scripts/splitlibraries_fastq.html/, accessed on 27 March 2023) [50,51] and the data are visualized in the form of a bar graph of bacterial flora composition and a heat map. By using the UPARSE algorithm (Uparse v7.0.1001; https://drive 5.com/uparse/, accessed on 26 March 2023) in QIIME and the SILVA database, gene sequences of the 16S rRNA were clustered into amplicon sequence variants (ASVs) at 97% similarity [52]. The 16S V4 region in the SILVA database was considered to construct the ASV table [52]. The whole-genome sequence data were submitted to the Bioproject of DDBJ Sequence Read Archive under accession numbers DRA017009 (experiment 1) and DRA017010 (experiment 2).

### 4.8. Statistical Analysis

Statistical analyses were performed by “Tukey–Kramer MRT”, and a different letter means significance at *p* < 0.05 via the statistical software package SPSS 16.0 (https://spss.software.informer.com/16.0, accessed on 3 May 2023). Mothur website was used to analyze the Shannon-Weiner diversity index (http://www.mothur.org/wiki/Shannon, accessed on 1 May 2023). Someka excel solutions was used to prepare a Venn diagram (https://www.someka.net/products/venn-diagram, accessed on 5 May 2023). Principal component analysis (PCA) analyses were performed using PAST 4.03 software (https://past.en.lo4d.com/windows, accessed on 10 May 2023). 

## 5. Conclusions

The diversity and abundance of endophytic bacterial communities should be studied for ensuring future crop growth and sustainability under constant environmental conditions. Our study revealed that insecticide treatment reduced the diversity of endophytic bacteria in *B. rapa*. In addition, experiment 2 revealed that increasing doses of dinotefuran decreased the diversity and abundance of endophytic bacteria. Changes in bacterial dominance and abundance were observed in both the culturable and non-cultivable experimental groups. *Bacillus* spp. was mostly abundant and dominant during the early stages of plant growth in both the insecticide treatment and control groups. This is a novel observation, and the identified bacteria may represent potential candidates for biodegradation studies on these insecticides in the future. Therefore, our findings suggest that insecticides play an adverse role in endophytic bacterial community enrichment especially at higher doses, which threatens future crop sustainability.

## Figures and Tables

**Table 1:** Impact of insecticide treatment on endophytic bacterial richness.

(A)				
Treatments	2nd Week		4th Week	
	Culturable	Non-Culturable	Culturable	Non-Culturable
C	9	41	14	217
N1	5	16	7	21
N2	7	19	11	32
N3	6	41	4	169
N4	7	31	6	42
**(B)**				
**Treatments**	**2nd Week**		**4th Week**	
	**Culturable**	**Non-Culturable**	**Culturable**	**Non-Culturable**
C	9	50	15	144
T1	7	31	6	42
T2	6	19	6	15
T3	5	16	4	9

## Data Availability

All data generated or analyzed during this study are included in this published article. In addition, sequencing data were deposited to DNA Data Bank of Japan (DDBJ).

## Supplementary Materials

The supporting information can be downloaded at: https://www.mdpi.com/article/10.3390/ijms242015306/s1.
