# Supplementary material for: Changes in the Endophytic Bacterial Community of Brassica rapa after Application of Systemic Insecticides
Source: Int J Mol Sci. 2023 Oct 18;24(20):15306. doi: 10.3390/ijms242015306 (PMC10607537; doi:10.3390/ijms242015306)
Supplement: Supplementary file 1 [file ijms-24-15306-s001.zip › ijms-2664516-supplementary.pdf]

Changes in the endophytic bacterial community of *Brassica rapa* after application of systemic insecticides

Md. Tareq Bin Salam, Ryota Kataoka

Supplementary data:

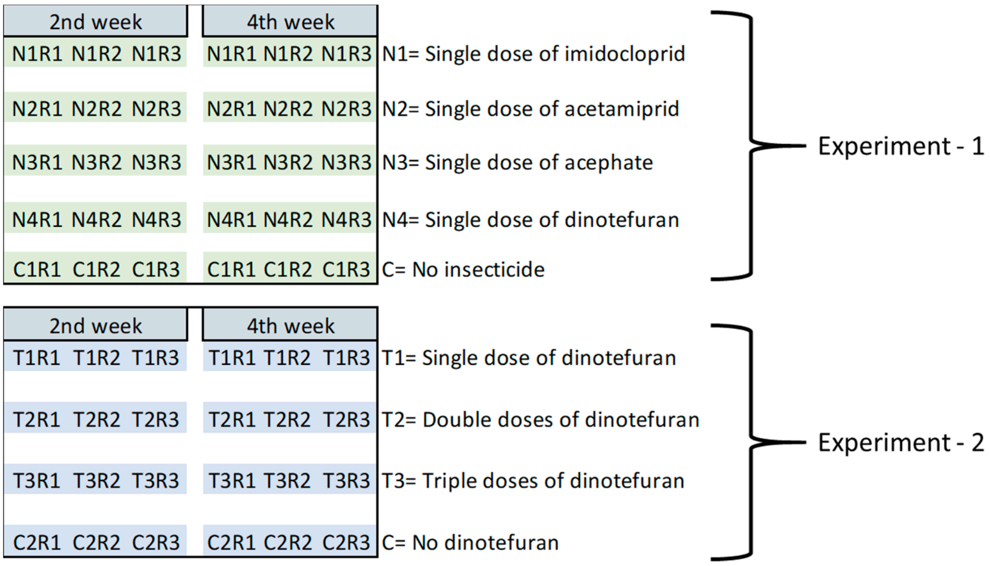

Figure S1: Layout of the experiment

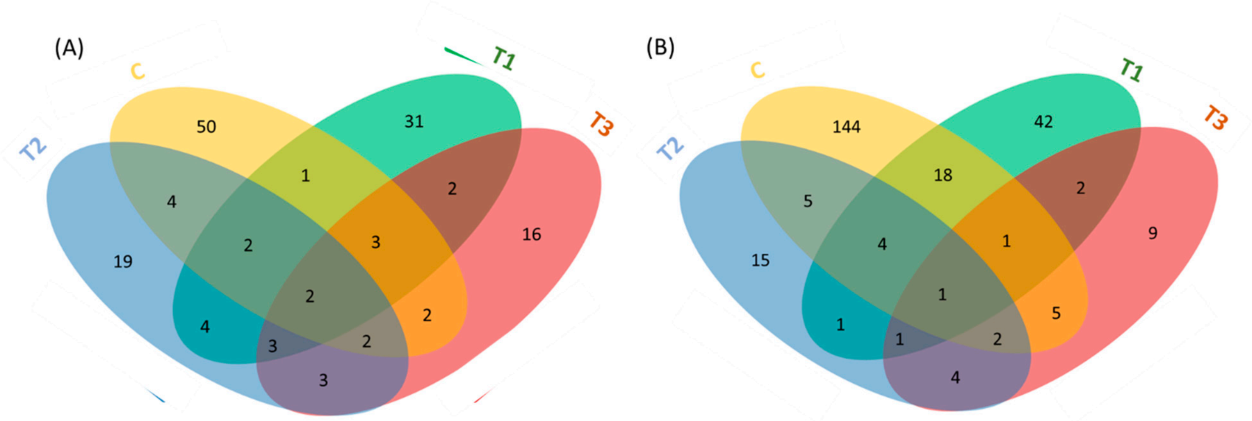

Figure S2: Venn- diagram represents the non-culturable bacterial richness along with bacterial similarities and differences among treatments at (A) 2nd week, (B) 4th week. Here, C, no dinotefuran application; T1, single dose of dinotefuran application; T2, double dose of dinotefuran application; and T3, triple dose of dinotefuran application.

Table S1: Frequency of culturable endophytic bacterial isolates, their nearest genera (>97% similarities) and their accession number from different treatments of systemic insecticides along with control

Treatment	Week	Type	Name of Bacterium	Relative frequency	Accession no.
Control	2nd week	1	*Brucella sp.*	15%	LC773881
		2	*Rhodococcus sp.*	15%	LC773882
		3	*Bacillus thuringiensis*	35%	LC773883
		4	*Curtobacterium sp.*	5%	LC773884
		5	*Burkholderia cenocepacia*	5%	LC773885
		6	*Methylobacterium sp.*	5%	LC773886
		7	*Paenarthrobacter nicotinovorans*	10%	LC773887
		8	*Microbacterium sp*	5%	LC773888
		9	*Bacillus subtilis*	5%	LC773889
	4th week	1	*Ralstonia sp.*	16%	LC773908
		2	*Burkholderia cenocepacia*	19%	LC773909
		3	*Bacillus thuringiensis*	6%	LC773910
		4	*Comamonadaceae bacterium*	3%	LC773911
		5	*Herbaspirillum chlorophenolicum*	3%	LC773912
		6	*Pseudomonas alcaligenes*	9%	LC773913
		7	*Sphingobium yanoikuyae*	3%	LC773914
		8	*Sphingomonas yantingensis*	6%	LC773915
		9	*Microbacterium sp*	6%	LC773916

		10	*Staphylococcus epidermidis*	6%	LC773917
		11	*Ralstonia sp.*	6%	LC773918
		12	*Sphingomonas koreensis*	6%	LC773919
		13	*Mitsuaria sp.*	6%	LC773920
		14	*Agrobacterium tumefaciens*	3%	LC773921
N1	2nd week	1	*Rhodococcus kroppenstedtii*	29%	LC773890
		2	*Bacillus sp. (in: firmicutes)*	29%	LC773891
		3	*Brucella pseudogrignonensis*	14%	LC773892
		4	*Nocardioides sp.*	14%	LC773893
		5	*Burkholderia cenocepacia*	14%	LC773894
	4th week	1	*Pseudomonas sp.*	25%	LC773922
		2	*Microbacterium sp.*	21%	LC773923
		3	*Agrobacterium pusense*	8%	LC773924
		4	*Microbacterium paraoxydans*	8%	LC773925
		5	*Paenarthrobacter nicotinovorans*	21%	LC773926
		6	*Variovorax sp.*	8%	LC773927
		7	*Duganella lactea*	8%	LC773928
N2	2nd week	1	*Curtobacterium sp.*	6%	LC773895
		2	*Brucella sp.*	31%	LC773896
		3	*Stenotrophomonas maltophilia*	13%	LC773897

		4	*Bacillus thuringiensis*	13%	LC773898
		5	*Nocardioides sp.*	19%	LC773899
		6	*Brucella pseudogrignonensis*	6%	LC773900
		7	*Microbacterium sp*	13%	LC773901
	4th week	1	*Labrys sp. (in: a-proteobacteria)*	25%	LC773929
		2	*Sphingomonas sp.*	8%	LC773930
		3	*Stenotrophomonas maltophilia*	21%	LC773931
		4	*Agrobacterium pusense*	4%	LC773932
		5	*Sphingobium yanoikuyae*	8%	LC773933
		6	*Mitsuaria chitosanitabida*	8%	LC773934
		7	*Bacillus subtilis*	4%	LC773935
		8	*Gottfriedia acidiceleris*	8%	LC773936
		9	*Microbacterium sp.*	4%	LC773937
		10	*Niabella yanshanensis*	4%	LC773938
		11	*Streptomyces griseorubiginosus*	4%	LC773939
N3	2nd week	1	*Paenarthrobacter nicotinovorans*	38%	LC773902
		2	*Burkholderia cenocepacia*	19%	LC773903
		3	*Curtobacterium sp.*	19%	LC773904
		4	*Bacillus thuringiensis*	6%	LC773905
		5	*Ochrobactrum teleogrylli*	13%	LC773906

		6	*Brucella pseudogrignonensis*	6%	LC773907
	4th week	1	*Pseudomonas sp.*	50%	LC773940
		2	*Ensifer adhaerens*	31%	LC773941
		3	*Microbacterium sp.*	13%	LC773942
		4	*Bacillus subtilis*	6%	LC773943
N4	2nd week	1	*Bacillus proteolyticus*	36%	LC773453
		2	*Ectobacillus panaciterrae*	7%	LC773454
		3	*Priestia aryabhattai*	14%	LC773455
		4	*Sphingomonas sp.*	14%	LC773456
		5	*Pseudomonas koreensis*	7%	LC773457
		6	*Paenibacillus glycanilyticus*	7%	LC773458
		7	*Paenibacillus sp.*	14%	LC773459
	4th week	1	*Bacillus subtilis*	33%	LC773460
		2	*Asticcacaulis taihuensis*	28%	LC773461
		3	*Pseudoxanthomonas sp.*	17%	LC773462
		4	*Priestia aryabhattai*	11%	LC773463
		5	*Stenotrophomonas maltophilia*	6%	LC773464
		6	*Paenibacillus glycanilyticus*	6%	LC773465

Table S2: Frequency of culturable endophytic bacterial isolates, their nearest genera (>97% similarities) and their accession number from different doses of dinotefuran treated plants along with control

Treatment	Week	Type	Name of Bacterium	Relative frequency	Accession no.
Control	2nd week	1	*Priestia aryabhattai*	25%	LC773429
		2	*Bacillus velezensis*	8%	LC773430
		3	*Paenibacillus sp.*	17%	LC773431
		4	*Bacillus tropicus*	8%	LC773432
		5	*Paenibacillus pocheonensis*	8%	LC773433
		6	*Paenibacillus glycanilyticus*	8%	LC773434
		7	*Bacillus subtilis*	8%	LC773435
		8	*Burkholderia cenocepacia*	8%	LC773436
		9	*Bacillus cereus*	8%	LC773437
	4th week	1	*Bacillus cereus*	21%	LC773438
		2	*Paenibacillus sp.*	15%	LC773439
		3	*Paenibacillus cineris*	9%	LC773440
		4	*Paenibacillus favisporus*	6%	LC773441
		5	*Neobacillus drentensis*	6%	LC773442
		6	*Paenibacillus glycanilyticus*	6%	LC773443
		7	*Bosea sp. (in: a-proteobacteria)*	3%	LC773444
		8	*Priestia aryabhattai*	6%	LC773445
		9	*Lysinibacillus xylanilyticus*	6%	LC773446
		10	*Bacillus thuringiensis*	6%	LC773447
		11	*Bacillus cereus*	3%	LC773448

		12	*Bacillus sp.*	6%	LC773449
		13	*Burkholderia cenocepacia*	3%	LC773450
		14	*Bacillus subtilis*	3%	LC773451
		15	*Paenibacillus rigui*	3%	LC773452
T1	2nd week	1	*Bacillus proteolyticus*	36%	LC773453
		2	*Ectobacillus panaciterrae*	7%	LC773454
		3	*Priestia aryabhattai*	14%	LC773455
		4	*Sphingomonas sp.*	14%	LC773456
		5	*Pseudomonas koreensis*	7%	LC773457
		6	*Paenibacillus glycanilyticus*	7%	LC773458
		7	*Paenibacillus sp.*	14%	LC773459
	4th week	1	*Bacillus subtilis*	33%	LC773460
		2	*Asticcacaulis taihuensis*	28%	LC773461
		3	*Pseudoxanthomonas sp.*	17%	LC773462
		4	*Priestia aryabhattai*	11%	LC773463
		5	*Stenotrophomonas maltophilia*	6%	LC773464
		6	*Paenibacillus glycanilyticus*	6%	LC773465
T2	2nd week	1	*Pseudomonas sp.*	27%	LC773466
		2	*Paenibacillus sp.*	18%	LC773467
		3	*Paenibacillus glycanilyticus*	18%	LC773468
		4	*Bacillus sp.*	9%	LC773469
		5	*Bacillus subtilis*	9%	LC773470
		6	*Priestia megaterium*	18%	LC773471

	4th week	1	*Priestia megaterium*	38%	LC773472
		2	*Paenibacillus sp.*	28%	LC773473
		3	*Bacillus velezensis*	17%	LC773474
		4	*Mesorhizobium sp.*	10%	LC773475
		5	*Sphingobium sp.*	3%	LC773476
		6	*Priestia aryabhattai*	3%	LC773477
T3	2nd week	1	*Margalitia shackletonii*	43%	LC773478
		2	*Nocardioides aromaticivorans*	29%	LC773479
		3	*Paenarthrobacter nitroguajacolicus*	14%	LC773480
		4	*Priestia aryabhattai*	7%	LC773481
		5	*Priestia megaterium*	7%	LC773482
	4th week	1	*Pseudomonas koreensis*	50%	LC773483
		2	*Priestia aryabhattai*	17%	LC773484
		3	*Bacillus pseudomycoides*	17%	LC773485
		4	*Bacillus thuringiensis*	17%	LC773486
